# Head, acetabular liner composition, and rate of revision and wear in total hip arthroplasty: a Bayesian network meta-analysis

**DOI:** 10.1038/s41598-023-47670-z

**Published:** 2023-11-21

**Authors:** Ricarda Merfort, Nicola Maffulli, Ulf Krister Hofmann, Frank Hildebrand, Francesco Simeone, Jörg Eschweiler, Filippo Migliorini

**Affiliations:** 1 Department of Orthopaedic, Trauma, and Reconstructive Surgery, RWTH University Hospital, 52074 Aachen, Germany; 2 Department of Medicine and Psicology, University La Sapienza, Rome, Italy; 3 Faculty of Medicine, School of Pharmacy and Bioengineering, Keele University, Thornburrow Drive, Stoke-on-Trent, England, UK; 4 Centre for Sports and Exercise Medicine, Barts and the London School of Medicine and Dentistry, Queen Mary University of London, Mile End Hospital, 275 Bancroft Road, London, E1 4DG England, UK; 5 Department of Orthopaedic and Trauma Surgery, Academic Hospital of Bolzano (SABES-ASDAA), Teaching Hospital of the Paracelsus Medical University, 39100 Bolzano, Italy

**Keywords:** Health care, Medical research, Pathogenesis, Risk factors

## Abstract

Total hip arthroplasty (THA) is a common procedure for patients suffering from hip pain e.g. from osteoarthritis, osteonecrosis, or hip fractures. The satisfaction of patients undergoing THA is influenced by the choice of implant type and material, with one key factor being the selection of the appropriate material combination for the bearing surface. In this Bayesian network meta-analysis, we investigated the impact of material combinations for the bearing surface on the longevity of hip implants. The wear penetration rate per year and the total wear penetration in the liner resulting from different material combinations, as well as the survival rate at last follow-up, were examined. We analyzed a total of 663,038 THAs, with 55% of patients being women. Mean patient age was 59.0 ± 8.1 years and mean BMI 27.6 ± 2.6 kg/m^2^. The combination of an aluminium oxide (Al_2_O_3_) head and an Al_2_O_3_ liner demonstrated the lowest wear penetration at last follow-up and the lowest rate of wear penetration per year. Additionally, the combination of a crosslinked polyethylene (XLPE) liner and a zircon oxide (ZrO_2_) head demonstrated the lowest rate of revision at last follow-up. These findings underscore the importance of careful material selection for hip implant bearing surfaces to optimize their longevity and patient satisfaction after THA.

## Introduction

Total hip arthroplasty (THA) is a well-established and successful procedure to treat patients with osteoarthritis or injuries of the hip or other forms of joint degeneration. With 233,537 cases, THA was one of the ten most commonly performed surgeries in Germany in 2021^1^. With life expectancy on the rise, the likelihood of a reoperation and even multiple reoperations after THA increases. In around 42% of THAs, the estimated survival time of the implant is lower than 25 years, and revision surgery is required^2^. Although hip implants vary in design, conventional hip implants consist of a stem that is fixed to the femur, an acetabular component fixed to the pelvis, a femoral head which is connected with the stem, and an insert for the acetabular component. The interaction between the femoral head and the insert is referred to as the bearing surface: this is where the joint actually moves and as such where friction and wear take place. In addition to other factors such as patient expectation, BMI, age, sex, comorbidities, length of hospital stay, and the type of surgical approach, the choice of the prosthesis design plays a crucial role for patient satisfaction^3–6^. Low wear rates and high survival rates are important factors for selecting prosthetic design and material.

The most common bearing surfaces utilized in THA include metal-on-polyethylene (MoP), metal-on-metal (MoM), ceramic-on-ceramic (CoC), and ceramic-on-polyethylene (CoP)^7^. The most commonly used metals are cobalt-chromium alloys (CoCr), stainless steel or oxidized zirconium (OxZr). Ceramic materials, such as aluminium oxide (Al_2_O_3_), zircon oxide (ZrO_2_) and alumina toughed zirconia (AMC/ZTA), are used for head and liner. Polyethylene liners can be made of conventional ultrahigh-molecular-weight polyethylene (UHMWPE), or by further crosslinking of UHMWPE to crosslinked polyethylene (XLPE), moderately cross-linked polyethylene (MXLPE), highly cross-linked polyethylene (HXLPE) and Vitamin E-infused HXLPE (HXLPE-VEPE).

Wear between the bearing surfaces is a complex phenomenon involving material characteristics, lubrication and friction^8^. Different wear phenomena, such as abrasion, adhesion, and tribocorrosion, can occur between the bearing surfaces and lead to material loss and debris production^8^. The production of wear particles can lead to pseudotumor formation and aseptic loosening, with bone loss as a consequence of biological reaction from abrasive particles^9, 10^. Apart from wear between the bearing surfaces, wear in hip implants can occur at modular junctions such as the taper and neck^11–13^ or between the acetabular component and liner^14^ from micromotion.

Wear in the bearing surface of the patient’s hip implant can be estimated on radiolographic images in different ways. One way is to determine the one-dimensional linear femoral head penetration. This can be accomplished measuring the centre difference of femoral head and liner, manually or -mostly used- by computer-assisted techniques^15^. Another method is to consider volumetric wear as the material loss in all three dimensions. During their lifetime, the wear rate of implants varies. Higher wear rates are observed in the running-in phase during the first one million (walking-)cycles, usually during the first 12 months from THA. Later, the wear coefficient decreases in the steady state phase^8^.

Relevant factors for implant survival are the surgeon, the patient, his/her activity, and the choice of implant. A number of meta-analyses have investigated wear and revision rates of different bearing surface materials, yet mostly focussing on one material or the comparison of two different material combinations^16–22^.

To support the choice of the material for the bearing surface of a hip replacement, we performed a Bayesian network meta-analysis where we looked into different material combinations of head and liner with respect to revision interval, total wear penetration and wear penetration per year. The following material combinations were studied: Al_2_O_3_–Al_2_O_3_, AMC/ZTA–AMC/ZTA, CoCr–AMC/ZTA, CoCr–CoCr, CPE/UHMPE–ZrO_2_, CPE/UHMPE–CoCr, CPE/UHMPE–Al_2_O_3_, CPE/UHMPE–OxZr, HXLPE–CoCr, HXLPE–Al_2_O_3_, HXLPE–ZrO_2_, HXLPE–AMC/ZTA, HXLPE–Stainless-Steel, HXLPE–VEPE–CoCr, HXLPE-VEPE–AMC/ZTA, MXLPE–CoCr, MXLPE–AMC/ZTA, XLPE–CoCr, XLPE–Al2O3, XLPE–OxZr, XLPE-VEPE–CoCr.

## Methods

### Eligibility criteria

All clinical investigations which compared two or more material combinations for head and inlay in THA were accessed. Only studies published in peer-reviewed journals were considered. According to the authors´ language capabilities, articles in English and German were eligible. Only studies with level I to IV of evidence, according to Oxford Centre of Evidence-Based Medicine^1^, were considered. Reviews, opinions, letters, and editorials were not considered. Animal studies, in vitro, biomechanics, computational, and cadaveric studies were not eligible. Missing quantitative data under the outcomes of interests warranted the exclusion of the study.

### Search strategy

This study was conducted according to the PRISMA extension statement for reporting of systematic reviews incorporating network meta-analyses of health care interventions: checklist and explanations^23^. The PICOT algorithm was preliminary pointed out:

- P (Problem): End stage hip OA;
- I (Intervention): THA;
- C (Comparison): Different material combinations of head and inlay;
- O (Outcomes): Rate of revision surgery, total wear penetration, wear penetration per year
- T (Timing): Minimum 12 months follow-up.

In September 2023, the following databases were accessed: PubMed, Scopus, Embase, Google Scholar, Cochrane. A time constraint was set from January 2000 to September 2023. The following matrix of keywords were used in each database to accomplish the search using the Boolean operator AND/OR: THA AND (hip OR arthroplasty OR replacement OR prosthesis) AND (metal OR ceramic OR alumina OR zirconia OR polyethylene OR steel) AND (wear OR revision). No additional filters were used in the databases search.

### Selection and data collection

Two authors (F.M. and R.M.) independently performed the database search. All the resulting titles were screened by hand and, if suitable, the abstract was accessed. The full-text of the abstracts which matched the topic of interest were accessed. If the full-text was not accessible or available, the article was not considered for inclusion. A cross reference of the bibliography of the full-text articles was also performed for inclusion. Disagreements were debated and mutually solved by the authors. In case of further disagreements, a third senior author (J.E.) took the final decision.

### Data items

Two authors (R. M. and F. M.) independently performed data extraction. The following data at baseline were extracted: author, year of publication and journal, length of the follow-up, number of patients with related mean age and BMI (Kg/m^2^). The following data were collected at last follow-up: inlay wear penetration (mm), inlay wear penetration per year (mm/year), rate of revision.

### Assessment of the risk of bias and quality of the recommendations

Two reviewers (U.K.H. and F.M.) evaluated the risk of bias of the extracted studies independently. The included studies were evaluated using the risk of bias of the software Review Manager 5.3 (The Nordic Cochrane Collaboration, Copenhagen)^24^. The following endpoints were evaluated: selection, detection, performance, attrition, reporting, and other bias.

### Synthesis methods

The statistical analyses were performed by one author (F.M.) following the recommendations of the Cochrane Handbook for Systematic Reviews of Interventions^25^. For descriptive statistics, mean and standard deviation were used. For baseline comparability, the IBM SPSS software was used. Comparability was assessed through the Analysis of Variance (ANOVA), with *P* > 0.1 considered satisfactory. The network analyses were made through the STATA/MP software (Stata Corporation, College Station, Texas, USA). Only studies which stated clearly the nature of the material of the component (head and/ or liner) were included in the analyses. An overview of the material combinations of head and liner included in the present Bayesian network meta-analysis is shown in Table 1.

**Table 1:** Material combinations of head and liner included in the present Bayesian network meta-analysis.

Liner	Head
Al_2_O_3_	Al_2_O_3_
AMC/ZTA	AMC/ZTA
CoCr	AMC/ZTA
CoCr	CoCr
CPE/UHMWPE	ZrO_2_
CPE/UHMWPE	CoCr
CPE/UHMWPE	Al_2_O_3_
CPE/UHMWPE	Stainless-Steel
CPE/UHMWPE	OxZr
HXLPE	CoCr
HXLPE	Al_2_O_3_
HXLPE	ZrO_2_
HXLPE	AMC/ZTA
HXLPE	Stainless-Steel
HXLPE-VEPE	CoCr
HXLPE-VEPE	AMC/ZTA
MXLPE	CoCr
MXLPE	AMC/ZTA
XLPE	CoCr
XLPE	Al_2_O_3_
XLPE	OxZr

The analyses were performed through the Stata routine for Bayesian hierarchical random-effects model analysis. Continuous variables were analysed through the inverse variance method, with the standardized mean difference (SMD) effect measure. Binary data were analysed through the Mantel–Haenszel method, with the Log Odd Ratio (LOR) effect measure. Edge, interval, and funnel plots were performed and analysed. The overall transitivity, consistency, and heterogeneity, as well as the size of the treatment effect of interest within-study variance, were evaluated. The overall inconsistency was evaluated through the equation for global linearity via the Wald test. In P_Wald_ values > 0.05, the null hypothesis could not be rejected, and the consistency assumption could be accepted at the overall level of each treatment. Confidence and percentile intervals (CI a d PrI, respectively) were each set at 95%.

### Ethical approval

This study complies with ethical standards.

## Results

### Study selection

**Figure 1:**
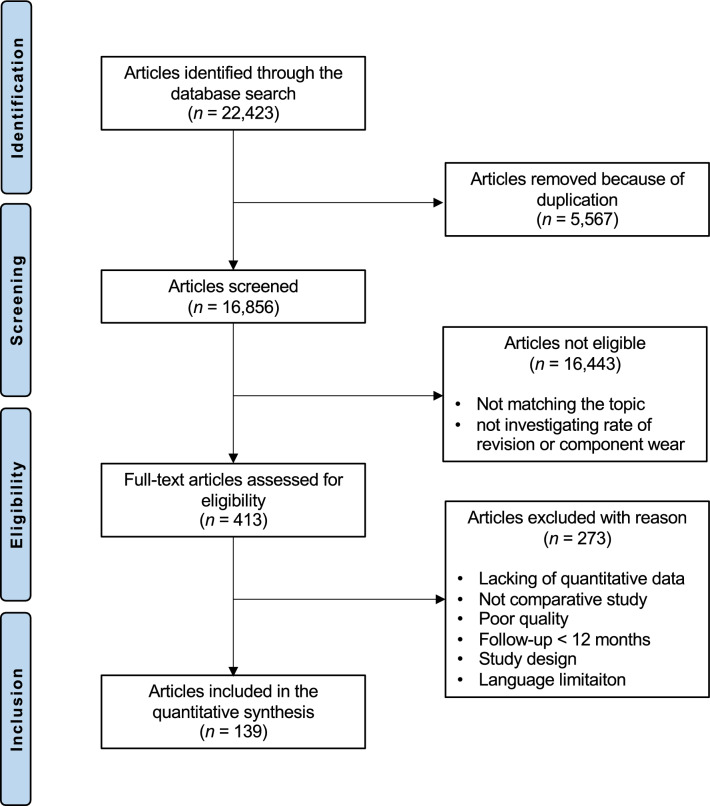
PRISMA flow chart of the literature search.

The initial databases search resulted in 22,423 articles. Of these, 5567 duplicates were excluded. After screening titles and abstracts 16,443 articles were excluded because they did not match the following eligibility criteria: not comparing two or more bearing material combinations, not mentioning rate of revision surgery or wear related values, no matching study design, not focusing on THA. Of the remaining 413 articles, another 274 were excluded because they did not report quantitative data for wear penetration, or rate of revision surgery, or the follow up time was shorter than 12 months. Finally, 139 studies were included in this review. The results of the literature search are shown in Fig. 1.

### Risk of bias assessment

**Figure 2:**
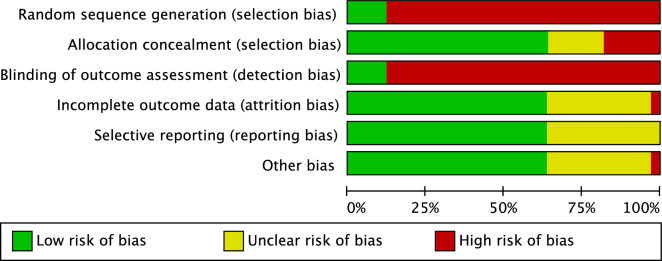
Cochrane risk of bias tool.

The Cochrane risk of bias tool was performed to investigate the risk of bias of the included studies. Given the number of retrospective studies included in the present investigation, the risk of selection bias was moderate. Few authors performed assessor blinding, leading to a moderate risk of detection bias. The risk of attrition and reporting biases was moderate, as was the risk of other bias. Concluding, the risk of bias graph evidenced a moderate quality of the methodological assessment (Fig. 2).

### Study characteristics

Data from 663,038 THAs were collected. 55% of patients were women. The mean patient age was 59.0 ± 8.1 years, the mean BMI was 27.6 ± 2.6 kg/m^2^. The mean length of follow-up was 87.9 ± 46.3 months. At baseline, no statistically significant difference was found in mean age, BMI, and mean length of follow-up (*P* > 0.5). The generalities and demographic and further basic data of the included studies are shown in Table 2.

**Table 2:** Generalities and patient baseline data of the included studies.

Author	Year	Design	Head	Liner	Patients (n)	Mean Age	Mean BMI	Women (%)	Follow-up (months)
Beauchamp et al.^64^	2021	Retrospective	CoCr	CoCr	13	58.1	28.6	0.14	60.4
			Alumina matrix composite/Zirconia toughened alumina	Alumina matrix composite/Zirconia toughened alumina	17	50.5	28.5	0.12	52.8
Vendittoli et al.^65^	2021	Prospective	Alumina oxide ceramic (Al_2_O_3_)	Alumina oxide ceramic (Al_2_O_3_)	71				252.0
			Stainless-Steel	Polyethylene	69				252.0
Busch et al.^66^	2020	RCT	Alumina oxide ceramic (Al_2_O_3_)	Polyethylene (HXLPE)	43	62.3	28.5	0.56	60.0
			Alumina oxide ceramic (Al_2_O_3_)	Polyethylene (HXLPE-VEPE)	51	62.3	28.5	0.54	60:0
Frisch et al.^67^	2020	Prospective	CoCr	CoCr	49	57.5	33.6	0.51	91.2
			Ceramic/Metal	Ceramic/Polyethylene	26	58.7	33.7	0.54	120.3
Kim et al.^68^	2020	Prospective	Alumina oxide ceramic (Al_2_O_3_)	Alumina oxide ceramic (Al_2_O_3_)	133	53.0	28.0	0.37	205.2
			Alumina oxide ceramic (Al_2_O_3_)	Polyethylene (HXLPE)	133	53.0	28.0	0.37	205.2
Kjærgaard et al.^59^	2020	RCT	CoCr	Polyethylene (HXLPE-VEPE)	24	65.0	28.0	0.21	
			CoCr	Polyethylene (HXLPE-VEPE)	29	63.0	29.0	0.31	
			CoCr	Polyethylene (XLPE)	30	64.0	28.0	0.36	
			CoCr	Polyethylene (XLPE)	33	61.0	27.0	0.42	
Massier et al.^69^	2020	Prospective	Alumina oxide ceramic (Al_2_O_3_)	Polyethylene (HXLPE-VEPE)	102	66.0		0.75	72.0
			Alumina oxide ceramic (Al_2_O_3_)	Polyethylene (CPE/UHMWPE)	97	65.0		0.66	72.0
Ong et al. ^70^	2020	Retrospective	Ceramic	Polyethylene	3620	58.0	22.6	0.61	37.2
			Metal	Polyethylene	9480			0.61	54.0
			Alumina matrix composite/Zirconia toughened alumina	Polyethylene (HXLPE)	163			0.91	70.8
Thoen et al.^71^	2020	RCT	CoCr	Polyethylene (HXLPE-VEPE)	37	58.0	28.5	0.46	
			CoCr	Polyethylene (MXLPE)	31	61.0	26.6	0.48	
Thompson et al.^72^	2020	Prospective	non-Metal	non-Metal	91	42.5			109.2
			CoCr	CoCr	30	53.0			109.2
van der Veen et al.^73^	2020	Retrospective	CoCr	CoCr	23	78.8		0.74	158.4
			CoCr	Polyethylene (CPE/UHMWPE)	33	78.7		0.79	162.0
Bryan et al.^74^	2019	Retrospective	CoCr	Polyethylene (HXLPE)	216	42.6	29.6		
			CoCr	Polyethylene (CPE/UHMWPE)	57	40.1	26.3		
Feng et al.^75^	2019	Prospective	Alumina matrix composite/Zirconia toughened alumina	Polyethylene (HXLPE)	77	59.0	23.2	0.43	86.4
			Alumina matrix composite/Zirconia toughened alumina	Alumina matrix composite/Zirconia toughened alumina	93	51.0	25.20	0.43	82.80
Galea et al.^76^	2019	Prospective	Alumina matrix composite/Zirconia toughened alumina	Polyethylene (HXLPE-VEPE)	39	66.1	27.2	0.56	
			Alumina matrix composite/Zirconia toughened alumina	Polyethylene (MXLPE)	34	62.6	28.3	0.59	
Sköldenberg et al.^77^	2019	Prospective	CoCr	Polyethylene (HXLPE-VEPE)	21	67.0	27.0	0.48	
			CoCr	Polyethylene (CPE/UHMWPE)	21	67.0	27.0	0.52	
Atrey et al. ^78^	2018	Prospective	Alumina oxide ceramic (Al_2_O_3_)	Alumina oxide ceramic (Al_2_O_3_)	28	41.5	26.7	0.50	180.0
			Alumina oxide ceramic (Al_2_O_3_)	Polyethylene (CPE/UHMWPE)	29	42.8	28.2	0.55	180.0
Galea et al.^79^	2018	Prospective	Alumina matrix composite/Zirconia toughened alumina	Polyethylene (HXLPE-VEPE)					60.0
			Alumina matrix composite/ Zirconia toughened alumina	Polyethylene (MXLPE)					60.0
			CoCr	Polyethylene (MXLPE)					60.0
Galea et al.^79^	2018	Prospective	CoCr	Polyethylene (HXLPE-VEPE)		59.0	28.1	0.62	60.0
			Alumina matrix composite/Zirconia toughened alumina	Polyethylene (HXLPE)					72.0
Higuchi et al.^80^	2018	Retrospective	CoCr	Polyethylene (HXLPE)	77	64.7	23.1	0.88	79.2
			Alumina oxide ceramic (Al_2_O_3_)	Alumina oxide ceramic (Al_2_O_3_)	105	55.9	23.0	0.81	80.4
Hopper et al.^81^	2018	Prospective	CoCr	Polyethylene (XLPE)	116	62.5	28.6	0.56	188.4
			CoCr	Polyethylene (CPE/UHMWPE)	114	62.0	27.9	0.50	176.4
Martin et al.^82^	2018	Prospective	Alumina matrix composite/Zirconia toughened alumina	Alumina matrix composite/Zirconia toughened alumina	42	60.0	26.4	0.14	94.0
			CoCr	CoCr	40	54.0	30.6	0.55	74.0
Morrison et al.^83^	2018	Prospective	yttria-stabilized zirconia YSZ	Polyethylene (CPE/UHMWPE)	20	81.7	26.2	0.70	139.0
			CoCr	Polyethylene (CPE/UHMWPE)	18	80.6	32.6	0.72	140.0
Peters et al.^84^	2018	Retrospective	Metal	Polyethylene (CPE/UHMWPE)	37,351				108.0
			Metal	Polyethylene (HXLPE)	32,867				108.0
			Ceramic	Polyethylene (CPE/UHMWPE)	40,109				108.0
			Ceramic	Polyethylene (HXLPE)	70,175				108.0
			Ceramic	Ceramic	17,625				108.0
			Oxidized zirconium (OxZr)	Polyethylene (UHMWPE and HXLPE)	11,785				108.0
Sharplin et al.^85^	2018	Retrospective	Ceramic	Ceramic	11,235			0.48	56.5
			Ceramic	Metal	474			0.37	56.5
			Composite Ceramic	Polyethylene (CPE/UHMWPE)	6833			0.50	63.2
			Composite Ceramic	Polyethylene (HXLPE)	14,382			0.48	62.9
			Metal	Metal	5989			0.36	54.1
			Metal	Polyethylene (CPE/UHMWPE)	35,647			0.59	72.1
			Metal	Polyethylene (HXLPE)	31,579	M: 68.6 W: 70.7		0.54	96.1
Teeter et al.^86^	2018	Retrospective	Ceramic	Polyethylene (HXLPE)	20	57.1	30.4	0.80	61.2
			CoCr	Polyethylene (HXLPE)	20	57.2	31.0	0.80	67.2
			Oxidized zirconium (OxZr)	Polyethylene (HXLPE)	18	59.9	31.0	0.44	62.4
			CoCr	Polyethylene (HXLPE)	18	60.1	35.2	0.44	64.8
Atrey et al.^87^	2017	RCT	CoCr	Polyethylene (HXLPE)	29				120.0
			CoCr	Polyethylene (CPE/UHMWPE)	34				120.0
			Ceramic	Ceramic	29				120.0
Borgwardt et al.^88^	2017	RCT	Zirconia (ZrO_2_)	Polyethylene (CPE/UHMWPE)	76	66.4		0.54	120.0
			CoCr	CoCr	72	68.2		0.58	120.0
			Zirconia (ZrO_2_)	Polyethylene (CPE/UHMWPE)	75	69.8		0.65	120.0
			Alumina oxide ceramic (Al_2_O_3_)	Alumina oxide ceramic (Al_2_O_3_)	76	69.1		0.75	120.0
Broomfield et al.^89^	2017	Prospective	CoCr	Polyethylene (CPE/UHMWPE)	27	68.0		0.45	146.4
			CoCr	Polyethylene (HXLPE)	27	67.0		0.53	146.4
Dahlstrand et al.^90^	2017	RCT	CoCr	CoCr	41	65.0	27.0	0.51	192.0
			CoCr	Polyethylene (CPE/UHMWPE)	44	67.0	27.0	0.54	192.0
Devane et al.^91^	2017	RCT	CoCr	Polyethylene (CPE/UHMWPE)	59	61.0		0.47	132.0
			CoCr	Polyethylene (HXLPE)	57	61.0		0.37	132.0
Gillam et al.^92^	2017	Retrospective	Metal (Large—Head)	Metal	231	M: 77.8 W: 80.2		0.46	
			Metal (Small—Head)	Metal	121	M: 77.3 W: 79.4		0.38	
			Metal	Polyethylene	3546	M: 82.3 W: 82.2		0.58	
			Metal (Articular Surface Replacement)	Metal	121	M: 81.6 W: 80.6		0.48	
Kawata, et al.^50^	2017	Prospective	Zirconia (ZrO_2_)	Polyethylene (CPE/UHMWPE)	26	60.0			
			Zirconia (ZrO_2_)	Polyethylene (HXLPE)	25	61.5			
			Zirconia (ZrO_2_)	Polyethylene (HXLPE)	23	62.6			
			Stainless-Steel	Polyethylene (HXLPE)	20	60.8			
Nebergall et al.^93^	2017	Prospective	Ceramic	Polyethylene (HXLPE-VEPE)	32	67.0	27.0	0.50	
			Ceramic	Polyethylene (MXLPE)	35	65.0	27.0	0.54	
Scemama et al.^94^	2017	Prospective	CoCr	Polyethylene (CPE/UHMWPE)	50	66.0	26.0	0.48	
			CoCr	Polyethylene (HXLPE-VEPE)	50	67.0	25.0	0.56	
Schouten et al.^95^	2017	Prospective	Alumina matrix composite/Zirconia toughened alumina	CoCr	36	62.0	30.0	0.50	60.0
			CoCr	CoCr	31	64.0	30.0	0.32	60.0
Takada et al.^58^	2017	Retrospective	Ceramic	Polyethylene (HXLPE)	54	60.1	22.5	0.89	63.6
			Ceramic	Polyethylene (HXLPE)	55	65.5	23.2	0.84	63.6
Teeter et al.^96^	2017	RCT	CoCr	Polyethylene (CPE/UHMWPE)	8	67.5	28.4		156.0
			CoCr	Polyethylene (HXLPE)	8	67.5	28.4		156.0
Tsukamoto et al.^97^	2017	Retrospective	CoCr	Polyethylene (HXLPE)	41	56.3		0.93	150.0
			CoCr	Polyethylene (CPE/UHMWPE)	38	57.9		0.89	156.0
Engh et al.^98^	2016	RCT	Alumina matrix composite/Zirconia toughened alumina	Alumina matrix composite/Zirconia toughened alumina	194	59.0	30.0	0.43	50.0
			CoCr—high carbid	CoCr	196	60.0	30.0	0.46	50:0
Hamai et al.^60^	2016	Retrospective	Zirconia (ZrO_2_)	Polyethylene (XLPE)	36	61.1		0.86	121.2
			Zirconia (ZrO_2_)	Polyethylene (XLPE)	36	60.7		0.86	121.2
Hanna et al.^99^	2016	Retrospective	CoCr	Polyethylene (CPE/UHMWPE)	89	56.8	30.7	0.51	158.4
			CoCr	Polyethylene (HXLPE)	88	55.6	30.0	0.90	157.2
Higuchi et al.^100^	2016	Retrospective	Alumina oxide ceramic (Al_2_O_3_)	Alumina oxide ceramic (Al_2_O_3_)	67	54.0	23.9	0.78	132.0
			CoCr	Polyethylene (HXLPE)	81	54.2	22.5	0.83	135.6
Petis et al.^101^	2016	Retrospective	CoCr	Polyethylene (HXLPE)	311	54.9	31.0	0.50	98.4
			Oxidized zirconium (OxZr)	Polyethylene (HXLPE)	311	54.8	30.9	0.50	93.6
Sato et al.^102^	2016	Retrospective	Ceramic	Polyethylene (CPE/UHMWPE)	110	60.3	20.4	0.85	228.0
			Ceramic	Polyethylene (CPE/UHMWPE)	73	59.8	22.0	0.85	241.2
Sillesen et al.^103^	2016	Retrospective	CoCr	Polyethylene (HXLPE-VEPE)	520	60.8	28.3	0.50	
			CoCr	Polyethylene (MXLPE)	457	62.3	28.5	0.50	
Garvin et al.^104^	2015	Prospective	CoCr	Polyethylene (HXLPE)	19	42.0	30.0		108.00
			Ceramic	Polyethylene (HXLPE)	34	42.0	30.0		108.00
Garvin et al.^104^	2015	Prospective	Oxidized zirconium (OxZr)	Polyethylene (HXLPE)	43	42.0	30.0	0.53	108.00
			not mentioned	Polyethylene (CPE/UHMWPE)	19	67.0			120.00
Glyn-Jones et al.^105^	2015	Prospective	not mentioned	Polyethylene (HXLPE)	20	68.0		0.45	120.00
			Alumina oxide ceramic (Al_2_O_3_)	Alumina oxide ceramic (Al_2_O_3_)	80				
Jassim, et al.^106^	2015	Prospective	CoCr	Polyethylene (HXLPE)	123	61.0		0.66	60.00
			Oxidized zirconium (OxZr)	Polyethylene (HXLPE)	121	63.0		0.56	60.00
			Oxidized zirconium (OxZr)	Polyethylene (CPE/UHMWPE)	124	63.0		0.56	60.00
Jonsson et al.^107^	2015	Prospective	CoCr	Polyethylene (CPE/UHMWPE)	30	69.0	27.0	0.67	
			Oxidized zirconium (OxZr)	Polyethylene (CPE/UHMWPE)	30	69.0	26.0	0.77	
			CoCr	Polyethylene (HXLPE)	30	70.0	27.0	0.67	
			Oxidized zirconium (OxZr)	Polyethylene (HXLPE)	30	70.0	27.0	0.73	
Karidakis et al.^108^	2015	Retrospective	Alumina oxide ceramic (Al_2_O_3_)	Polyethylene (CPE/UHMWPE)	45				
			Alumina oxide ceramic (Al_2_O_3_)	Polyethylene (XLPE)	46				
			Oxidized zirconium (OxZr) (28 mm head)	Polyethylene (XLPE)	48				
			Oxidized zirconium (OxZr) (32 mm head)	Polyethylene (XLPE)	49				
Keeney et al.^109^	2015	Retrospective	CoCr	Polyethylene (CPE/UHMWPE)	84	40.4	28.8	0.43	
			CoCr	Polyethylene (HXLPE)	89	40.3	27.7	0.58	
Langlois et al.^110^	2015	Prospective	CoCr	Polyethylene (HXLPE)	50	66.4	24.4	0.55	
			CoCr	Polyethylene (CPE/UHMWPE)	50	66.4	24.4	0.55	
Pang et al.^111^	2015	Retrieval	CoCr	Polyethylene (HXLPE)	13	61.0	32.0	0.62	
			CoCr	Polyethylene (CPE/UHMWPE)	13	66.0	32.0	0.62	
Shareghi et al.^112^	2015	Prospective	CoCr	Polyethylene (XLPE-VEPE)	38	58.0	25.0	0.42	
			CoCr	Polyethylene (XLPE)	32	58.0	27.0	0.53	
Varnum et al.^113^	2015	Registry	Ceramic	Ceramic	1773	59.0		0.47	60.0
			Metal	Polyethylene	9323	65.0		0.51	46.8
Epinette et al.^114^	2014	Retrospective	Alumina oxide ceramic (Al_2_O_3_)	Polyethylene (HXLPE)	228	68.7	28.1	0.66	125.9
			Alumina oxide ceramic (Al_2_O_3_)	Alumina oxide ceramic (Al_2_O_3_)	447	68.0	27.4	0.68	134.9
Furnes et al.^115^	2014	Registry	Metal	Metal	14,373			0.52	
			Metal	Polyethylene (HXLPE)	10,699			0.42	
Lübbeke et al.^116^	2014	Prospective	CoCr	CoCr	883	63.1	27.4		92.0
			Alumina oxide ceramic (Al_2_O_3_)	Polyethylene (CPE/UHMWPE)	2458	72.0	27.0		124.0
Morison et al.^117^	2014	RCT	CoCr	Polyethylene (CPE/UHMWPE)	21	50.6	30.3	0.48	81.6
			CoCr	Polyethylene (HXLPE)	23	53.7	27.9	0.48	81.6
			Oxidized zirconium (OxZr)	Polyethylene (CPE/UHMWPE)	21	52.4	27.1	0.36	81.6
			Oxidized zirconium (OxZr)	Polyethylene (HXLPE)	22	51.2	29.3	0.55	81.6
Parsons et al.^118^	2014	Retrospective	CoCr	Polyethylene (CPE/UHMWPE)	27	64.7		0.26	90.6
			Alumina oxide ceramic (Al_2_O_3_)	Polyethylene (CPE/UHMWPE)	36	57.8		0.56	118.8
			CoCr	CoCr	18	59.0		0.44	100.8
Topolovec et al.^119^	2014	Retrieval	CoCr—low carbid	CoCr—low carbid	26	68.0		0.92	
			Stainless-steel	Polyethylene (CPE/UHMWPE)	12	74.0		0.67	
			Stainless-steel	Polyethylene	587	69.4		0.76	
Dahl et al.^120^	2013	Retrospective	CoCr	Polyethylene (CPE/UHMWPE)	23	60.0		0.74	120.0
			Alumina oxide ceramic (Al_2_O_3_)	Polyethylene (CPE/UHMWPE)	20	64.0		0.55	120.0
Desmarchelier et al.^121^	2013	RCT	Metal	Metal	125	63.7	25.4	0.68	100.5
			Alumina oxide ceramic (Al_2_O_3_)	Alumina oxide ceramic (Al2O3)	125	59.6	25.8	0.45	109.8
Fukui et al.^122^	2013	Retrospective	Zirconia (ZrO_2_)	Polyethylene (HXLPE)	36	56.7	23.1	0.94	124.8
			Zirconia (ZrO_2_)	Polyethylene (CPE/UHMWPE)	20	53.0	22.7	0.80	127.2
García-Rey et al.^123^	2013	Prospective	CoCr	Polyethylene (HXLPE)	42	67.4		0.57	
			Stainless-Steel	Polyethylene (CPE/UHMWPE)	41	61.1		0.54	
Hasegawa et al.^124^	2013	Prospective	Yttria stabilized zirconia	Polyethylene (HXLPE)	23	64	24.1	0.91	84.0
			Alumina stabilized zirconia	Polyethylene (HXLPE)	68	57	23.2	0.91	84.0
Huang et al.^125^	2013	Registry	Metal	Metal	1118	62.0		0.52	38.4
			Metal	Polyethylene (HXLPE)	1286	68.0		0.56	51.6
Kim et al.^126^	2013	Prospective	Alumina oxide ceramic (Al_2_O_3_)	Alumina oxide ceramic (Al_2_O_3_)	100	45.3		0.50	148.8
			Alumina oxide ceramic (Al_2_O_3_)	Polyethylene (HXLPE)	100	45.3		0.50	148.8
Nakashima et al.^127^	2013	Retrospective	Zirconia (ZrO_2_)	Polyethylene (CPE/UHMWPE)	62	62.0	23.9	0.70	156.6
			Zirconia (ZrO_2_)	Polyethylene (HXLPE)	69	61.8	24.3	0.82	137.8
Vendittoli et al.^128^	2013	RCT	Alumina oxide ceramic (Al_2_O_3_)	Alumina oxide ceramic (Al2O3)	69	56.8	27.3	0.45	147.6
			Metal	Polyethylene (CPE/UHMWPE)	71	54.9	28.2	0.58	147.6
Wang et al.^129^	2013	Retrospective	Alumina oxide ceramic (Al_2_O_3_)	Polyethylene (CPE/UHMWPE)	22	51.5		0.50	120.0
			CoCr	Polyethylene (CPE/UHMWPE)	22	51.5		0.50	120.0
Bozic et al.^130^	2012	Registry	Metal	Metal	49,646			0.58	
			Metal	Polyethylene	93,929			0.64	
			Ceramic	Ceramic	5252			0.59	
Cai et al.^131^	2012	RCT	Alumina matrix composite/Zirconia toughened alumina	Alumina matrix composite/Zirconia toughened alumina	51	42.1	24.6	0.51	39.7
			Alumina oxide ceramic (Al_2_O_3_)	Polyethylene (CPE/UHMWPE)	62	42.0	24.8	0.56	40.3
D'Antonio et al.^132^	2012	Prospective	Alumina oxide ceramic (Al_2_O_3_)	Alumina oxide ceramic (Al_2_O_3_)	144	54.2	27.9	1.00	123.6
			CoCr	Polyethylene (CPE/UHMWPE)	72	54.2	27.9	1.00	123.6
Engh et al.^133^	2012	RCT	CoCr	Polyethylene (HXLPE)	116	62.5	28.6	0.56	
			CoCr	Polyethylene (CPE/UHMWPE)	114	62.0	27.9	0.50	
Hanna et al.^134^	2012	Prospective	CoCr—high carbid	Polyethylene (CPE/UHMWPE)	22	72.0	28.7	0.77	
			CoCr—high carbid	CoCr—high carbid	27	68.0	28.1	0.78	
Johanson et al.^135^	2012	Prospective	CoCr	Polyethylene (CPE/UHMWPE)	27	56.0		0.44	
			CoCr	Polyethylene (HXLPE)	25	55.0		0.52	
Kadar et al.^49^	2012	Registry	CoCr	Polyethylene (CPE/ UHMWPE)	5232	73.0		0.73	74.4
			CoCr	Polyethylene (CPE/UHMWPE)	3195	73.0		0.74	94.8
			Alumina oxide ceramic (Al2O3)	Polyethylene (CPE/UHMWPE)	448	74.0		0.70	75.6
			Zirconia (ZrO_2_)	Polyethylene (CPE/UHMWPE)	275	64.0		0.65	121.2
Nikolaou et al.^136^	2012	RCT	CoCr	Polyethylene (CPE/UHMWPE)	36	52.6	28.7	0.50	60.0
			CoCr	Polyethylene (HXLPE)	32	55.1	32.6	0.56	60.0
			Ceramic	Ceramic	34	52.0	28.2	0.50	60.0
Porat et al.^137^	2012	Retrospective	Ceramic	Ceramic	1757	50.0	35.0	0.40	
			Metal	Metal	1589	58.0	31.4	0.48	
Sato et al.^52^	2012	Retrospective	Zirconia (ZrO_2_)	Polyethylene (CPE/UHMWPE)	40	59.6		0.63	145.2
			Alumina oxide ceramic (Al2O3)	Polyethylene (CPE/UHMWPE)	24	59.6		0.56	145.2
			Zirconia (ZrO_2_)	Polyethylene (HXLPE)	275	61.8		0.85	73.2
			Alumina oxide ceramic (Al_2_O_3_)	Polyethylene (HXLPE)	72	61.8		0.85	73.2
			CoCr	Polyethylene (HXLPE)	20	61.8		0.85	73.2
Schouten et al.^138^	2012	RCT	Alumina matrix composite/ Zirconia toughened alumina	CoCr	41	61.5	29.0	0.45	
			CoCr	CoCr	36	63.8	29.0	0.36	
Amanatullah et al.^139^	2011	Prospective	Alumina oxide ceramic (Al_2_O_3_)	Alumina oxide ceramic (Al_2_O_3_)	196	50.4	29.6	0.36	
			Alumina oxide ceramic (Al_2_O_3_)	Polyethylene (HXLPE)	161	54.7	28.0	0.43	
Mall et al.^140^	2011	Retrospective	CoCr	Polyethylene (CPE/UHMWPE)	50	43.2			72.2
			CoCr	Polyethylene (HXLPE)	48	46.5			99.5
Malviya et al.^141^	2011	RCT	CoCr	CoCr	50	63.9	28.6	0.62	
			CoCr	Polyethylene (CPE/UHMWPE)	50	64.9	29.4	0.54	
Molli et al.^142^	2011	Retrospective	CoCr	CoCr	1589	57.4	31.4	0.47	47.5
			CoCr	Polyethylene (HXLPE/MXLPE)	779	70.3	29.1	0.66	42.9
Orradre Burusco et al.^143^	2011	Prospective	Alumina oxide ceramic (Al_2_O_3_)	Polyethylene (HXLPE)	50	65.4	25.5	0.36	64.8
			Alumina oxide ceramic (Al_2_O_3_)	Polyethylene (CPE/UHMWPE)	57	67.6	25.6	0.40	69.6
Thomas et al.^144^	2011	Prosective	CoCr	Polyethylene (HXLPE)	22	68.0		0.55	84.0
			CoCr	Polyethylene (CPE/UHMWPE)	22	67.0		0.50	84.0
Bascarevic et al.^145^	2010	RCT	Alumina oxide ceramic (Al_2_O_3_)	Alumina oxide ceramic (Al_2_O_3_)	82	53.9	26.7	0.79	51.0
			CoCr	Polyethylene (HXLPE)	75	55.6	27.8	0.68	50.0
Hamilton et al.^146^	2010	RCT	Alumina matrix composite/Zirconia toughened alumina	Alumina matrix composite/Zirconia toughened alumina	177	56.4		0.49	31.1
			Alumina matrix composite/Zirconia toughened alumina	Polyethylene (HXLPE)	87	57.3		0.46	31.5
Huddleston et al.^147^	2010	Prospective	CoCr	Polyethylene (CPE/UHMWPE)	45	57.0	27.1	0.26	128.4
			CoCr	Polyethylene (CPE/UHMWPE)	43	60.0	25.4	0.43	120.0
Lewis et al.^148^	2010	RCT	Alumina oxide ceramic (Al_2_O_3_)	Polyethylene (CPE/UHMWPE)	23	42.8	28.2		120.0
			Alumina oxide ceramic (Al_2_O_3_)	Alumina oxide ceramic (Al2O3)	23	41.5	26.7		120.0
Lombardi et al.^149^	2010	RCT	Alumina matrix composite/Zirconia toughened alumina	Alumina matrix composite/Zirconia toughened alumina	64	57.0		0.45	73.0
			Alumina oxide ceramic (Al_2_O_3_)	Polyethylene (CPE/UHMWPE)	45	60.0		0.47	72.0
Nakahara et al.^53^	2010	Prospective	Zirconia (ZrO_2_)	Polyethylene (HXLPE)	47	57.5	23.5	0.81	80.4
			CoCr	Polyethylene (HXLPE)	47	56.9	23.5	0.87	79.2
Beksaç et al.^150^	2009	Retrospective	CoCr	Polyethylene (HXLPE)	41	50.0	28.0	0.43	63.6
			CoCr	Polyethylene (CPE/UHMWPE)	41	53.0	30.0	0.43	63.6
Calvert et al.^151^	2009	RCT	CoCr	Polyethylene (HXLPE)	60	62.5		0.45	
			CoCr	Polyethylene (CPE/UHMWPE)	59	61.0		0.59	
Geerdink et al.^152^	2009	RCT	CoCr	Polyethylene (CPE/UHMWPE)	26	64.0	28.0	0.43	96.0
			CoCr	Polyethylene (HXLPE)	22	64.0	28.0	0.35	96.0
Hernigou et al.^153^	2009	Retrospective	Alumina oxide ceramic (Al_2_O_3_)	Alumina oxide ceramic (Al_2_O_3_)	28	55.0			240.0
			Alumina oxide ceramic (Al_2_O_3_)	Polyethylene	28	55.0			240.0
Ise et al.^154^	2009	RCT	Zirconia (ZrO_2_)	Polyethylene (CPE/UHMWPE)	26	60.0		0.96	48.5
			Zirconia (ZrO_2_)	Polyethylene (HXLPE)	25	61.6		0.94	45.6
			Zirconia (ZrO_2_)	Polyethylene (HXLPE)	23	62.7		1.00	44.8
			Stainless steel	Polyethylene (HXLPE)	20	60.9		0.94	48.8
Kawate et al.^155^	2009	RCT	Zirconia (ZrO_2_)	Polyethylene (HXLPE)					
			CoCr	Polyethylene (HXLPE)					
Kim et al.^156^	2009	Retrospective	Alumina oxide ceramic (Al_2_O_3_)	Alumina oxide ceramic (Al_2_O_3_)	100	45.3	23.0	0.34	67.2
			Alumina oxide ceramic (Al_2_O_3_)	Polyethylene (HXLPE)	100	45.3	23.0	0.34	67.2
Rajadhyaksha et al.^157^	2009	Retrospective	CoCr	Polyethylene (HXLPE)	27	60.3	27.6	0.32	71.0
			CoCr	Polyethylene (CPE/UHMWPE)	27	62.0	28.1	0.44	75.0
Sexton et al.^158^	2009	Retrospective	Ceramic	Ceramic	20,627	68.1		0.55	
			Ceramic	Polyethylene	14,001	68.1		0.55	
			Metal	Metal	12,208	68.1		0.55	
			Metal	Polyethylene	62,437	68.1		0.55	
Stilling et al.^159^	2009	Retrospective	Zirconia (ZrO_2_)	Polyethylene (CPE/UHMWPE)	36	53.5		0.15	58.0
			CoCr	Polyethylene (CPE/UHMWPE)	33	51.5		0.42	58.0
			Zirconia (ZrO_2_)	Polyethylene (CPE/UHMWPE)	54	44.2		0.11	85.2
			CoCr	Polyethylene (CPE/UHMWPE)	54	44.2		0.11	85.2
Capello et al.^160^	2008	RCT	Alumina oxide ceramic (Al_2_O_3_)	Alumina oxide ceramic (Al_2_O_3_)	93	53.2	27.6	0.34	96.0
			Alumina oxide ceramic (Al_2_O_3_)	Alumina oxide ceramic (Al_2_O_3_)	92	55.1	28.3	0.33	100.8
			CoCr	Polyethylene (CPE/UHMWPE)	93	53.7	28.1	0.40	103.2
			Alumina oxide ceramic (Al_2_O_3_)	Alumina oxide ceramic (Al_2_O_3_)	174	51.8	28.2	0.31	78.0
García-Rey et al.^161^	2008	RCT	Stainless steel	Polyethylene (CPE/UHMWPE)	45	60.6			66.3
			CoCr	Polyethylene (HXLPE)	45	62.5			66.3
Miyanishi et al.^162^	2008	Retrospective	Zirconia (ZrO_2_)	Polyethylene (HXLPE)	95	67.0	24.7	0.83	27.6
			Zirconia (ZrO_2_)	Polyethylene (CPE/UHMWPE)	20	61.0	24.8	0.79	50.4
Digas et al.^163^	2007	Prospective	CoCr (cemented)	Polyethylene (HXLPE)	28	55.0		1.00	
			CoCr (cemented)	Polyethylene (CPE/UHMWPE)	27	55.0		1.00	
			CoCr (hybrid)	Polyethylene (HXLPE)	23	48.0		0.66	
			CoCr (hybrid)	Polyethylene (CPE/ UHMWPE)	23	48.0		0.66	
Kawanabe et al.^164^	2007	Prospective	Alumina oxide ceramic (Al_2_O_3_)	Polyethylene (CPE/UHMWPE)	46	58.1		0.88	80.4
			Zirconia (ZrO_2_)	Polyethylene (CPE/UHMWPE)	50	58.3		0.94	64.8
Kim et al.^165^	2007	Prospective	Alumina oxide ceramic (Al_2_O_3_)	Alumina oxide ceramic (Al_2_O_3_)	50	51.0		0.24	57.6
			Alumina oxide ceramic (Al_2_O_3_)	Polyethylene (CPE/UHMWPE)	50	51.0		0.24	57.6
Röhrl et al.^166^	2007	Retrospective	CoCr	Polyethylene (CPE/UHMWPE)	20	70.0		0.40	60.0
			CoCr	Polyethylene (HXLPE)	10	58.0		0.40	72.0
Triclot et al.^167^	2007	RCT	CoCr	Polyethylene (HXLPE)	33	67.9	26.5	0.48	59.5
			CoCr	Polyethylene (XLPE)	34	70.1	26.4	0.41	59.8
Vendittoli et al.^168^	2007	RCT	CoCr	Polyethylene (CPE/UHMWPE)	69	56.8		0.45	79.0
			Alumina oxide ceramic (Al_2_O_3_)	Alumina oxide ceramic (Al_2_O_3_)	71	54.9		0.58	79.0
Bragdon et al.^169^	2006	Prospective	CoCr	Polyethylene (HXLPE)	41	60.3			45.0
			CoCr	Polyethylene (HXLPE)	12	60.3			45.0
			CoCr	Polyethylene (CPE/UHMWPE)	70	60.3			45.0
Engh et al.^170^	2006	Prospective	CoCr	Polyethylene (HXLPE)	116	62.5	28.6	0.56	68.4
			CoCr	Polyethylene (CPE/UHMWPE)	114	62.0	27.9	0.50	68.4
Geerdink et al.^171^	2006	Prospective	CoCr	Polyethylene (CPE/UHMWPE)	54	63.0	27.0		56.4
			CoCr	Polyethylene (HXLPE)	45	64.0	28.0		56.4
Kraay et al.^172^	2006	RCT	CoCr	Polyethylene (CPE/UHMWPE)	30	68.9		0.65	51.7
			Zirconia (ZrO_2_)	Polyethylene (CPE/UHMWPE)	27	69.5		0.74	51.2
Oonishi et al.^173^	2006	Prospective	Alumina oxide ceramic (Al_2_O_3_)	Polyethylene (HXLPE)	70	61.0			28.0
			Alumina oxide ceramic (Al_2_O_3_)	Polyethylene (CPE/UHMWPE)	73	61.0			28.0
Seyler et al.^174^	2006	Retrospective	Alumina oxide ceramic (Al_2_O_3_)	Alumina oxide ceramic (Al_2_O_3_)	79	45.2	27.8	0.23	50.4
			Alumina oxide ceramic (Al_2_O_3_)	Alumina oxide ceramic (Al_2_O_3_)	79	46.5	29.8	0.22	58.8
			CoCr	Polyethylene (CPE/UHMWPE)	26	44.0	28.0	0.24	61.2
			CoCr	Polyethylene (CPE/ UHMWPE)	26	44.8	30.2	0.24	49.2
D'Antonio et al.^175^	2005	Retrospective	CoCr	Polyethylene (HXLPE)	56	57.4	26.9	0.49	58.8
			CoCr	Polyethylene (CPE/UHMWPE)	53	52.9	27.5	0.42	63.6
Dorr et al.^176^	2005	Prospective	CoCr	Polyethylene (HXLPE)	37	60.2		0.54	60.0
			CoCr	Polyethylene (CPE/UHMWPE)	37	65.1		0.54	60.0
Krushell et al.^177^	2005	Retrospective	CoCr	Polyethylene (HXLPE)	40	68.7	27.9	0.53	47.7
			CoCr	Polyethylene (CPE/ UHMWPE)	40	69.5	28.2	0.53	49.5
Liang et al.^178^	2005	Retrospective	Alumina oxide ceramic (Al_2_O_3_)	Polyethylene (CPE/UHMWPE)	45	58.0		0.89	74.4
			Zirconia (ZrO_2_)	Polyethylene (CPE/UHMWPE)	51	58.0		0.92	62.4
Manning et al.^179^	2005	Prospective	CoCr	Polyethylene (CPE/UHMWPE)	111	57.0	25.6	0.44	
			CoCr	Polyethylene (HXLPE)	70	60.9	25.9	0.50	44.0
Röhrl et al.^180^	2005	Prospective	CoCr	Polyethylene (CPE/UHMWPE)	20	70.0		0.40	24.0
			Zirconia (ZrO_2_)	Polyethylene (CPE/UHMWPE)	20	67.0		0.75	24.0
			CoCr	Polyethylene (HXLPE)	10	58.0		0.40	36.0
Sonny Bal et al.^181^	2005	Prospective	Alumina oxide ceramic (Al_2_O_3_)	Alumina oxide ceramic (Al_2_O_3_)	250	54.9		0.45	24.0
			Alumina oxide ceramic (Al_2_O_3_)	Polyethylene (CPE/UHMWPE)	250	60.9		0.53	24.0
Digas et al.^182^	2004	RCT	CoCr	Polyethylene (HXLPE)	27	48.0		0.63	
			CoCr	Polyethylene (CPE/UHMWPE)	27	48.0		0.63	
			CoCr	Polyethylene (HXLPE)	23	55.0		0.57	
			CoCr	Polyethylene (CPE/UHMWPE)	26	57.0		0.46	
Dorr et al.^183^	2004	Prospective	CoCr (cemented)	CoCr	153	69.0			60.0
			Zirconia (ZrO_2_) (cemented)	Polyethylene (CPE/UHMWPE)	148	67.0			60.0
			CoCr (uncemented)	CoCr	158	51.0			60.0
			Zirconia (ZrO_2_) ( uncemented)	Polyethylene (CPE/UHMWPE)	156	52.0			60.0
Jacobs et al.^184^	2004	Prospective	CoCr	CoCr	97	53.3		0.52	46.8
			CoCr	Polyethylene (MXLPE)	74	55.7		0.33	42.0
Hopper et al.^185^	2003	Retrospective	CoCr	Polyethylene (HXLPE)	78	58.7			37.2
			CoCr	Polyethylene (CPE/UHMWPE)	50	60.3			36.0
			CoCr	Polyethylene (HXLPE)	48	60.3			34.8
			CoCr	Polyethylene (CPE/UHMWPE)	50	61.0			33.6
Martell et al.^186^	2003	RCT	CoCr	Polyethylene (HXLPE)	24	60.0	30.6		27.6
			CoCr	Polyethylene (CPE/UHMWPE)	22	55.0	27.6		27.6
Pabinger et al.^187^	2003	RCT	CoCr	CoCr	31			0.39	24.0
			Alumina oxide ceramic (Al_2_O_3_)	Polyethylene (CPE/UHMWPE)	28			0.43	24.0
Taeger et al.^188^	2003	Prospective	Titanium. diamond-like-carbid (DLC) coated	Polyethylene (CPE/UHMWPE)	101	59.6		0.50	110.4
			Alumina oxide ceramic (Al_2_O_3_)	Polyethylene (CPE/UHMWPE)	101	57.0		0.63	110.4
D'Antonio et al.^189^	2002	Prospective	Alumina oxide ceramic (Al_2_O_3_)	Alumina oxide ceramic (Al2O3)	172	53.0		0.34	35.1
			Alumina oxide ceramic (Al_2_O_3_)	Alumina oxide ceramic (Al2O3)	177	53.0		0.36	35.2
			CoCr	Polyethylene (CPE/UHMWPE)	165	53.0		0.40	33.6
Kim et al.^190^	2001	Prospective	CoCr (22 mm)	Polyethylene (CPE/UHMWPE)	35	39.9		0.17	
			CoCr (28 mm)	Polyethylene (CPE/UHMWPE)	35	39.9		0.17	
			Zirconia (ZrO_2_) (22 mm)	Polyethylene (CPE/UHMWPE)	35	39.9		0.17	
			Zirconia (ZrO_2_) (28 mm)	Polyethylene (CPE/UHMWPE)	35	39.9		0.17	
Lombardi et al.^191^	2001	Prospective	CoCr	Polyethylene (CPE/UHMWPE)	72	48.9	28.7	0.24	39.5
			CoCr	CoCr	78	49.3	29.1	0.26	38.8
Pitto et al.^192^	2000	Prospective	Alumina oxide ceramic (Al_2_O_3_)	Polyethylene (CPE/UHMWPE)	25	62.0		0.67	60.0
			Alumina oxide ceramic (Al_2_O_3_)	Alumina oxide ceramic (Al_2_O_3_)	25	60.0		0.60	60.0

*RCT* randomised controlled trial; *CoCr* Cobalt-Chrome.

### Synthesis of results

**Figure 3:**
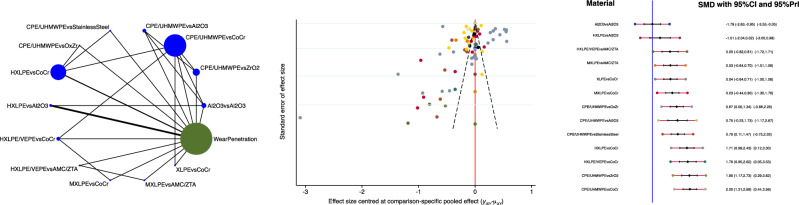
From left to the right: edge, funnel and interval plots of the comparison: overall wear penetration.

**Figure 4:**
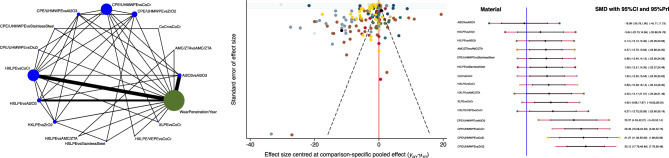
From left to the right: edge, funnel and interval plots of the comparison: wear penetration per year.

The combination of Al_2_O_3_ head and Al_2_O_3_ liner demonstrated the lowest wear penetration at last follow-up (Fig. 3) and the lowest rate of wear penetration per year (Fig. 4).

**Figure 5:**
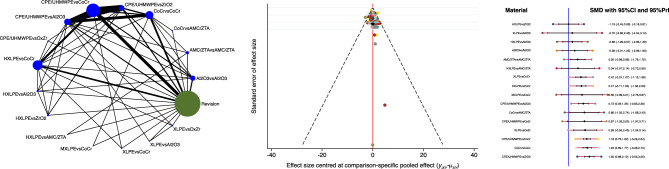
From left to the right: edge, funnel and interval plots of the comparison: rate of revision at last follow-up.

The combination of HXLPE head and ZrO_2_ demonstrated the lower rate of revision at last follow-up (Fig. 5). The equation of global linearity found no statistically significant inconsistency in all comparisons.

## Discussion

The choice of the best hip implant design and material of the bearing surface is crucial for patient satisfaction and longevity of the prosthesis. Different factors must be taken into account when choosing the best material combination for each patient. An important factor for the choice of the bearing surface biomaterial is wear, which remains a major problem in the long run leading to potentially aseptic loosening, pseudotumor formation, and pain. This network meta-analysis was conducted to compare the currently used material combinations for hip implant bearing surface regarding wear penetration, yearly penetration rate and revision surgeries.

In this Bayesian network meta-analysis, the combination of Al_2_O_3_ head and Al_2_O_3_ liner demonstrated the lowest wear penetration at last follow-up as well as the lowest rate of wear penetration per year. On the other hand, the combination of a HXLPE liner and ZrO_2_ head demonstrated the lowest rate of revision at last follow-up. Mean age, mean BMI, and mean length of the follow-up had no significant influence on wear behaviour and revision rate.

In general, bearing surfaces in hip implants can be distinguished in hard on soft bearings (with a polymeric material used for the liner and the hard femoral head) and in hard on hard (MoM or CoC) bearings. Given the hardness difference of the articulating partners, hard on hard bearings show lesser and smaller wear particles than hard on soft bearings^7, 26, 27^.

Wear modes in a tribological system depend on its structure, kinematic interactions, and the combination of wear phenomena. Wear modes are dynamic, and can change over time. Wear modes can be distinguished in normal wear (mode 1), wear occurring due to bearing surfaces articulating against non-bearing surfaces (mode 2), three-body wear (mode 3), and two non-bearing surfaces wearing against each other (mode 4). In the presence of hard wear particles, particularly, polyethylene wear increases. Harder materials result in a reduced contribution of third-body wear to overall wear^28, 29^.

CoC bearings have been used in THA for a long time given their biocompatibility, high wear resistance and chemical durability^7^. Additionally, CoC bearing combinations have the advantage to produce smaller and inert debris compared to other bearing types, leading to harmless wear to the human body. For this reason, they are generally considered a good choice for young patients^30^. The first generations of alumina ceramics had a high risk of fracture, which was later reduced by improving their manufacturing process^31^. Despite further improvements, ceramics as bearing surfaces still have weaknesses such as bearing noise and reduced toughness, which led to the development of advanced material combinations, such as AMC/ZTA, for use as bearing materials. Biomechanical studies have shown that AMZ/ZTA ceramics exhibit lower wear rates under extreme conditions compared to Al_2_O_3_^32, 33^. Nonetheless, our network meta-analysis found that Al_2_O_3_ ceramics had the lowest wear penetration rate per year and the least amount of wear at last follow up. This could possibly be explained by the fact that the latest material such as AMZ/ZTA is newer on the market and the average study duration is thus potentially shorter. We only included studies with a minimum duration of 12 months in our analysis; nevertheless, shorter study durations may overestimate debris and wear given the influence of running-in effects^17^.

Despite its good wear resistance, the Al_2_O_3_–Al_2_O_3_ combination did not exhibit the lowest revision rate in this meta-analysis. One major disadvantage of an Al_2_O_3_ combination are the disturbing noises which are associated with vibrations of the femoral implant system^34, 35^. Compared to MoP or MoM bearings, fracture of ceramic heads and liner still remains a major disadvantage for CoC bearings^34^. A study based on the Norwegian Arthroplasty Register found a 3.6 times higher occurrence of ceramic fracture in COC bearings compared to COP bearings. Furthermore, there was an elevated risk of fractures observed in Alumina ceramics compared to AMC heads^36^. Revision for ceramic fracture is of particular concern, as it can lead to catastrophic failures and severe complications because of third body wear caused by ceramic fragments^37, 38^. Additionally, the use of CoC bearings is expensive and requires an exquisite surgical insertion technique to avoid chipping off from contact surfaces^39^.

In this study, the combination of HXLPE liner and ZrO_2_ head demonstrated the lowest rate of revision at last follow-up. National registries are an important tool to compare revision rates of different material combinations. In the Australian Orthopaedic Association National Joint Replacement Registry (AOA) in 2022 Ceramised Metal head on XLPE liner exibit the lowest 10-year revision rate followed by ceramic head on XLPE liner, which, however, has the lowest 20-year revision rate with 6.8%. 20 year data for ceramised metal head on XLPE liner are not available yet^40^. The National Joint Registry (NJR) of England and Wales in 2022 reports ceramic on polyethylene to have the lowest 15 year revision rates for all fixation types^41^. The German Arthroplasty Registry (EPRD) registered the lowest 6-year-revision rate for CoC bearings for elective THA. Nevertheless, ceramic on HXLPE bearings were, with 49.2%, the most frequently used bearing type in Germany in 2021^42^. Regarding NJR data in England and Wales, MOP is still the most commonly used bearing with decreasing tendency, while the use of CoP bearings increases^41^. Crosslinked polyethylene is listed as the most commonly used polyethylene type, with 97.2% in 2021 in Australia^40^. In general, low revision rates for CoP and CoHXLPE are mentioned across all the registries.

The German registry classifies polyethylene into different degrees of crosslinking such as UHMWPE, MXLPE, and HXLPE, whereas the NJR only considers polyethylene as a single category. Similarly, the materials of the heads are divided only into broad categories of metal and ceramic or partly ceramised metals by the NJR. As a result, it is not possible to conduct a detailed analysis of the material properties in registry studies. Additionally, in registries, implant combinations are selected for patients based on individual characteristics, making comparisons between implant combinations highly susceptible to bias. Systematic reviews and meta-analyses have been conducted to overcome these limitations. A few exceptions aside^20^, most review studies only offer analyses of two or three material combinations^19, 43^.

We performed a comprehensive Bayesian network meta-analysis investigating more than 600,000 THA with 23 different material combinations. As mentioned, in registry studies, CoP bearings exhibit low revision rates. Biomechanical studies found improved wear behaviour for HXLPE compared to PE, which should also entail a longer lifetime^15, 44^. Zirconia as material for hip implants head has promising properties. In 2001, however, the largest manufacturer of zirconia femoral heads recalled their products for problems with thermal processing associated with some batches producing higher fracture rates, leading to a loss of confidence in zirconia as a reliable orthopaedic biomaterial^45, 46^. ZrO_2_ hip implant heads are also mentioned to be prone to aging^47^. Nevertheless, ZrO_2_ is widely used in dental applications^48^. A registry study in 2012 stated that ZrO_2_ heads are inferior to metal heads regarding revision rate at 12 years^49^. Of note, most studies evaluating ZrO_2_ on HXLPE bearing surfaces included in this network meta-analysis were performed in Japan^50–53^. Demographic characteristics could thus influence the results of this study. Nevertheless, the positive results for ZrO_2_ heads observed in the present network meta-analyses may prompt surgeons to rethink their attitude towards this material. However, only few studies investigated the survival rate of zirconia in the last few years.

The present study has several limitations that should be considered when interpreting the results. First, the influence of the head diameter, the fixation technique of stem and cup as well as the orientation of the cup and liner were not analysed. A high inclination angle can cause an increase in liner wear^54^. The head diameter of the prosthesis is an important factor that can affect the performance of the prosthesis, especially regarding the risk of dislocation^55, 56^. A larger head diameter can lead to increased volumetric wear in polyethylene cups, while linear wear remains consistent^57–59^. From our analyses, we cannot tell whether certain materials were preferably used in specific sizes. Future studies should consider the influence of head diameter in their analyses. Second, other types of head designs such as dual mobility bearings or hip resurfacing were not explicitly described. Although we subdivided polyethylene into different categories based on the descriptions used in the studies (CPE/UHMWPE, XLPE, HXLPE, MXLPE, HXLPE-VEPE), there could be differences arising from different manufacturing techniques such as annealing and remelting of the polyethylene or amount of crosslinking^60^. Currently, different treatments, including irradiation and melting, irradiation and annealing, sequential irradiation with annealing, irradiation followed by mechanical deformation, and irradiation and stabilization with vitamin E are available^61^. Irradiating UHMWPE results in cross-linking between the molecular chains, which improves the mechanical and tribological properties of this cross-linked PE^62^. The offset of that is that crosslinking affects the mechanical properties of UHMWPE, usually resulting in a decrease in toughness, stiffness, and hardness of the polymer^63^. Despite that effect, cross-linked UHMWPE is presently the standard of care.

In addition to randomized controlled studies, prospective and retrospective studies were included in this meta-analysis to provide additional data, leading to a moderate risk of bias. Prospective and retrospective studies have a higher risk of bias than randomized controlled trials because they may not use random allocation to balance potential confounding variables between treatment groups. In addition, the quality of the included studies varied, with some studies having a high risk of bias or unclear methodological quality. Nevertheless, a patient and case specific implant choice has to consider patient factors such as age, activity level, and weight, surgical technique, and cost in addition to wear rate and revision rate. Additionally, further design criteria are mandatory to be taken into account, including the fixation technique of the cup and stem within the bone.

## Conclusion

The combination of an Al_2_O_3_ head and an Al_2_O_3_ liner showed the lowest wear penetration at last follow-up, as well as the lowest rate of wear penetration per year. On the other hand, the combination of ZrO_2_ head ad HXLPE liner exhibited the lowest rate of revision at last follow-up.

## Data Availability

The datasets generated during and/or analysed during the current study are available throughout the manuscript.
